# Full-Genome Sequences of Alphacoronaviruses and Astroviruses from Myotis and Pipistrelle Bats in Denmark

**DOI:** 10.3390/v13061073

**Published:** 2021-06-04

**Authors:** Christina M. Lazov, Graham J. Belsham, Anette Bøtner, Thomas Bruun Rasmussen

**Affiliations:** 1 Department of Biotechnology and Biomedicine, Technical University of Denmark, 2800 Kongens Lyngby, Denmark; chmari@vet.dtu.dk; 2 Department of Veterinary and Animal Sciences, University of Copenhagen, 1870 Frederiksberg, Denmark; grbe@sund.ku.dk (G.J.B.); aneb@sund.ku.dk (A.B.); 3 Department of Virus and Microbiological Special Diagnostics, Statens Serum Institut, 2300 Copenhagen, Denmark

**Keywords:** virus excretion, RNA viruses, insect viruses, virus taxonomy

## Abstract

Bat species worldwide are receiving increased attention for the discovery of emerging viruses, cross-species transmission, and zoonoses, as well as for characterizing virus infections specific to bats. In a previous study, we investigated the presence of coronaviruses in faecal samples from bats at different locations in Denmark, and made phylogenies based on short, partial ORF1b sequences. In this study, selected samples containing bat coronaviruses from three different bat species were analysed, using a non-targeted approach of next-generation sequencing. From the resulting metagenomics data, we assembled full-genome sequences of seven distinct alphacoronaviruses, three astroviruses, and a polyomavirus, as well as partial genome sequences of rotavirus H and caliciviruses, from the different bat species. Comparisons to published sequences indicate that the bat alphacoronaviruses belong to three different subgenera—i.e., *Pedacovirus*, *Nyctacovirus*, and *Myotacovirus*—that the astroviruses may be new species in the genus *Mamastrovirus*, and that the polyomavirus could also be a new species, but unassigned to a genus. Furthermore, several viruses of invertebrates—including two *Rhopalosiphum padi* (aphid) viruses and a Kadipiro virus—present in the faecal material were assembled. Interestingly, this is the first detection in Europe of a Kadipiro virus.

## 1. Introduction

Bat species (order *Chiroptera*) are some of the most taxonomically diverse mammals, and with over 1400 recognized species (out of a total of around 6500 mammalian species worldwide), they are exceeded only by the number of rodent species [1]. Bats are found widely around the world, and fulfil important roles in ecosystems such as pollination, seed dispersal, regulation of insect populations, and fertilization [2]. In Europe, 51 species of bats have been described; all of them are protected by law, and 17 of these species in the family *Vespertilionidae* have ranges that include Denmark, in Scandinavia [3]. Bats have, in recent years, been the focus of research for the discovery of new viruses, since it has been found that spillover events from bats form the basis of many emerging infectious diseases in animals and humans [4]. Interestingly, many viruses are seemingly sustained in bat populations without causing disease. Reasons for this have been attributed to the unique immune system of bats, in which constitutively high levels of interferons are expressed, slowing down viral infections, while low antibody titers may be permissive of persistent infections with intermittent shedding [5,6]. Naturally, studies of bat viruses typically focus on those that have emerged as human pathogens, including coronaviruses, lyssaviruses, paramyxoviruses, filoviruses, and reoviruses [7,8]; this is reflected in the distribution of known virus sequences from bats [9]. Another approach has been to sequence and taxonomically assign the entire bat virome, e.g., all nucleic acids present in faecal samples [10,11,12,13,14,15]. Typically, these studies discover many viruses, but have low coverage of individual viruses, and mammalian, invertebrate, fungal, and bacterial viruses will all be present in the data.

Coronaviruses (CoVs), of the family *Coronaviridae* in the order *Nidovirales*, are enveloped viruses with positive-sense, single-stranded RNA genomes of around 27–31 kb [16]. Bats are regarded as a natural reservoir of coronaviruses in the genera *Alphacoronavirus* and *Betacoronavirus* [17]. Within these two genera, a number of important pathogens of animals and humans are known, e.g., porcine epidemic diarrhoea virus (PEDV), murine hepatitis virus (MHV), Middle East respiratory syndrome (MERS)-related coronavirus, severe acute respiratory syndrome (SARS) coronavirus and, most notably, the SARS-CoV-2 virus [18]. This virus, responsible for the COVID-19 pandemic, may have originated from bats [19,20,21,22,23], although the precise origin and possible intermediate hosts have not yet been identified. Likewise, the four endemic human coronaviruses (HCoVs)—OC43, 229E, NL63, and HKU1—have possible origins in bats and rodents [24,25,26].

Reports of coronaviruses in bats from the Nordic countries are limited. Previously, partial coronavirus ORF1b sequences from five different bat species in Denmark were reported [27] and, more recently, full-genome sequences of two separate coronaviruses—from *Myotis daubentonii* and *Myotis brandtii*—as well as other partial bat coronavirus (BtCoV) sequences, have been determined [28]. Still missing are full-genome sequences of coronaviruses from other bat species in the Nordic countries. In this study, we present assembled whole-genome sequences (termed full-genome or full-length sequences) of coronaviruses from *Myotis daubentonii*, *Myotis dasycneme*, and *Pipistrellus pygmaeus* in Denmark, tentatively placing them within three different subgenera within the genus *Alphacoronavirus*.

As a consequence of the non-targeted sequencing approach taken to obtain the coronavirus data, we gained additional information about the genome sequences of other viruses present within the bat faecal samples. This enabled us to assemble whole-genome sequences of three astroviruses and a polyomavirus, together with partial genome sequences of caliciviruses and rotaviruses. In addition to these viruses of vertebrate hosts, genomes were assembled of three virus taxa infecting arthropods—namely, *Rhopalosiphum padi* virus, Rosy apple aphid virus, and Kadipiro virus—as well as two picornavirus genomes of unknown host range, provisionally named Basavirus and bat picornavirus. The genome sequences of the dicistrovirus *Rhopalosiphum padi virus* [29] and the reovirus Kadipiro virus [30] were selected for further characterization, based on the overwhelming number of sequence reads derived from the samples. The astroviruses were selected for characterization due to the novelty of the genome sequences. The family *Astroviridae* comprises the genera *Mamastrovirus* (infecting mammals) and *Avastrovirus* (infecting birds) [31]. Astroviruses are highly prevalent—but understudied—infectious agents of bats [32], and few full-length sequences are publicly available.

## 2. Materials and Methods

The sample material for sequencing was nucleic acid extracted from selected bat faecal samples collected in 2018, or from samples collected earlier (2014–2016), from different regions in Denmark, as described previously [27], which had scored positive in pan-CoV RT-qPCR assays (Table 1). Selection was based on the level of coronavirus RNA in the samples (with Ct values as low as possible) and the distinct bat species and year of collection. Briefly, bat faecal samples were homogenized in Eagle’s medium, and the nucleic acids were extracted using a MagNA Pure 96 system (Roche, Basel, Switzerland) and then tested using up to three different pan-CoV real-time RT-qPCR assays, as previously described [27].

The nucleic acid samples (as described above) were treated with DNAse I (Invitrogen, Carlsbad, CA, USA) according to the manufacturer’s protocol, and double-stranded cDNA was generated using a Superscript III first-strand synthesis kit (Invitrogen) and a NEBNext mRNA second-strand synthesis kit (New England Biolabs, Ipswich, MA, USA). The reactions were purified using the GeneJET PCR purification kit (Thermo Scientific, Vilnius, Lithuania) with elution in 2 aliquots of nuclease-free water (30 µL each) at 60 °C.

The samples were sequenced at the DTU Multi-Assay Core (DMAC) in Kongens Lyngby, Denmark. In brief, the samples were dried in a Savant SpeedVac™ vacuum concentrator (Thermo Scientific, Waltham, MA, USA) and then resuspended in 5 μL nuclease-free water in order to increase their concentration. Libraries were generated using the Nextera XT DNA library Preparation Kit (Illumina Inc, San Diego, CA, USA) and checked on the 2100 Bioanalyzer system with the High-Sensitivity DNA kit (Agilent Technologies, Glostrup, Denmark). Note that the DNA content was often too low to pass the quality control (Supplementary Table S1), but the analyses were still continued. Finally, the libraries were run on the MiSeq platform using the Reagent Kit v3 600 bp (Illumina).

The raw reads were submitted to the online taxonomic investigation tool Kaiju version 1.7.3 [33], and the results were checked for the presence and number of reads matching coronaviruses and other viruses.

The raw read data were checked for quality using FastQC version 0.11.9 [34] and imported into Geneious Prime version 2019.2.3 to 2021.1.1 [35] for data processing. The reads were trimmed using the BBDuk plugin version 1.0 [36], and the trimmed reads were rechecked with FastQC (Supplementary Table S1).

The trimmed reads were assembled de novo in Geneious Prime using SPAdes version 3.13.0 [37] for metagenomic data with error correction and automatic k-mer choice, or with the MIRA plugin version 1.1.1 and automatic settings for small datasets below 500,000 reads. On the basis of the Kaiju analysis, the resulting contig sequences were mapped to reference sequences from GenBank [38] for bat coronaviruses and the other viruses assembled in the study. If one full-genome-length contig was identified, the trimmed reads were mapped to it, and a consensus sequence was extracted. If several partial-genome-length contigs were present, a reference sequence was used as a scaffold for the contigs, the trimmed reads were mapped to it, and a consensus sequence was generated. For mapping the reads, 5–10 iterations were used, with varying stringency—e.g., number of mismatches, ambiguities and length of gaps—allowed depending on the data, judged by visual inspection of the resulting contig. Occasionally, two or more substantially different de novo contigs would overlap, or multiple reads with identical nucleotide (nt) differences would map to the same de novo contig. In both cases, these observations indicated the presence of multiple virus variants in the sample. For these cases of mixed, closely-related virus populations, very stringent mappings were performed in order to assemble the predominant virus genome, followed by mapping of the unused reads in the dataset to assemble the variant genome(s). A similar strategy was applied when no suitable reference sequence was available and full-genome de novo contigs were not generated, either due to low coverage or other reasons. The reads were mapped to a partial genome de novo contig, and the unused reads were repeatedly mapped to the consensus sequence (Supplementary Table S2).

The consensus sequences were aligned with the de novo contig sequences and annotated using the closest related reference sequences from the ICTV Master Species List [39], in order to establish and appropriately trim the 5′ and 3′ termini of the newly assembled genomes, and to identify the ORFs.

Alignments were made with MUSCLE version 3.8.425 [40] or the progressive Mauve plugin version 1.1.3 [41] in Geneious Prime using type species reference sequences, and with the closest matching sequences identified using BLASTn. MUSCLE alignments of full-genome sequences, specific genes, or individual genome segments were used for generation of either maximum likelihood (ML) or neighbour-joining (NJ) phylogenetic trees, according to the normal practice for the particular virus family. ML trees based on Le–Gascuel and discrete gamma models (LG + G) were constructed from initial BioNJ trees with 100 bootstrap replicates, estimated proportions of invariable sites, and optimizations for tree topology, branch length, and rate using the PhyML plugin version 2.2.4 [42]. Geneious NJ phylogenetic trees based on the Jukes–Cantor genetic distance model were constructed with 1000 bootstrap replicates.

## 3. Results

### 3.1. Bat Coronaviruses

Bat coronavirus sequence reads (ranging from 1800 to 140,000 reads) were found in all seven samples selected for this study (see Table 1 and Table 2). Full-length genomes were assembled from the three different species of bats: five genomes were from four samples from *M. daubentonii* (two different viruses were obtained from sample 21164-6 in dataset D32-7), one genome from *M. dasycneme*, and one full-length plus one partial genome from *P. pygmaeus* (Table 3). The BtCoV genomes from *M. daubentonii* and *M. dasycneme* were assembled de novo using SPAdes, which, with one exception, produced a single contig covering the entire genome. For the *M. daubentonii* sample, containing two different BtCoVs, three overlapping partial genome contigs were produced by SPAdes, containing two different spike genes—this was the most divergent gene between the two BtCoVs in the sample. For the *P. pygmaeus* samples, one genome was assembled de novo into one full-length contig using MIRA, while the other genome was only partially assembled using an iterative reference mapping strategy due to the relatively low number of BtCoV reads (i.e., 1800) in this dataset (Supplementary Table S2).

**Figure 1:**
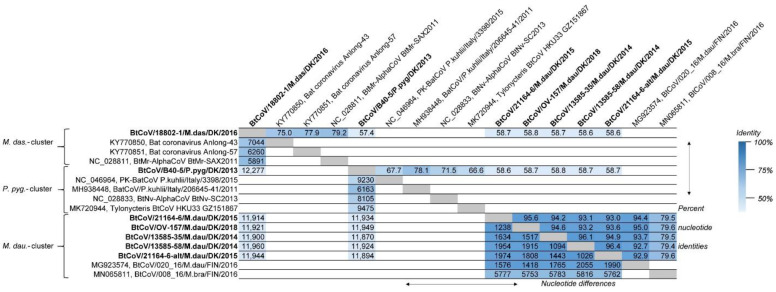
Comparison of full-length genomes of bat coronaviruses (BtCoVs) to one another, and to the closest matching full-length sequences from GenBank, after aligning the sequences with MUSCLE. The grouping of sequences corresponds to the clustering seen in the phylogenetic tree (Figure 2). The numbers in the upper-right part of the table are the percentage nucleotide identities between the sequences, while the lower-left part shows the actual number of nucleotide differences between the sequences. The heat map shading indicates degree of identity, with increased similarity shown by darker colours. The incomplete genome sequence BtCoV/7542-55/P.pyg/DK/2014 was not included in this analysis. The new sequences reported here are marked in bold text.

The full-length Danish BtCoV genomes were aligned and compared to one another, and to their closest BLASTn matches, for distance investigations between the nucleotide sequences (Figure 1). It was apparent that the BtCoVs from the three different bat species had low similarity between species (from 57.4 to 58.8% identity, corresponding to 12,277–11,870 nucleotide differences between the complete genomes). The BtCoV from *M. dasycneme* was most similar to the reference sequence NC_028811 from *M. pilosus* (also called *M. ricketti)*, with 79.2% identity and 5891 nucleotide differences, while the full-length BtCoV from *P. pygmaeus* was most similar to the genome from *P. kuhlii* (MH938448), with 78.1% identity and 6163 nucleotide differences. A much higher degree of similarity was found between the five BtCoV sequences from *M. daubentonii*, with a minimum of 93.0% and a maximum of 96.4% identity corresponding to 1974 and 1026 nucleotide differences, respectively. Interestingly, the lowest identity was found between the two BtCoVs from sample 21164-6. The Finnish CoV sequence (MG923574) from *M. daubentonii* was very similar to the Danish *M. daubentonii* CoV sequences (between 92.7% and 95.0% identity corresponding to 2055 and 1418 nucleotide differences, respectively).

The Danish BtCoV sequences were also aligned with the closest resembling reference sequences from BLAST using the progressive Mauve plugin in Geneious. The Mauve alignment showed no evidence for recombination or rearrangements.

**Figure 2:**
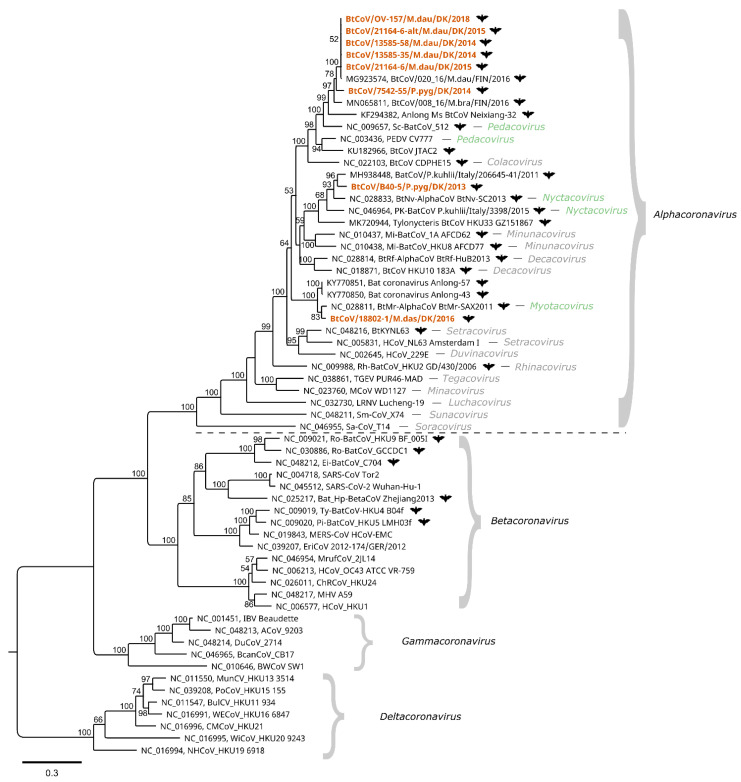
Phylogenetic tree of bat coronavirus sequences from Denmark. The sequences reported here (highlighted in orange) were compared to the closest matching sequences from GenBank and at least one ICTV exemplar isolate of each species of the *Orthocoronavirinae* subfamily. Bat coronavirus sequences are indicated with a bat symbol. The tree was generated using the PhyML maximum likelihood method with 100 bootstrap iterations on MUSCLE-aligned concatenated amino acid sequences predicted from the partial ORF1ab nt sequences (conserved regions of nsp5, nsp12, and nsp13), with a total length of 1224 amino acids. Only bootstrap values above 50 are displayed on the nodes. The evolutionary model for the tree was LG + G. The ICTV reference sequences have all been assigned to subgenera within the genera *Alphacoronavirus*, *Betacoronavirus*, *Gammacoronavirus*, and *Deltacoronavirus*. Clustering of the Danish bat coronavirus sequences indicates that they belong to the same subgenera as the reference sequences in the genus *Alphacoronavirus*, within the subgenera *Pedacovirus*, *Nyctacovirus*, and *Myotacovirus* (highlighted in green).

To further characterize the Danish BtCoVs, the ORF1ab and Spike protein coding sequences were translated in silico. For the Spike protein, an amino acid alignment was made with reference sequences including SARS-CoV-1, SARS-CoV-2, and PEDV. As for the PEDV Spike protein, no furin cleavage sites (motif RXXR) were detected in the bat coronaviruses, except in the cytoplasmic tail of the S2 (with the motif RGPR located at a position corresponding to a.a. 1367–1370 of PEDV Acc. No. NC_003436). For the ORF1ab sequences, an alignment was made of concatenated, conserved regions of certain non-structural proteins (nsps)—namely, nsp5, nsp12, and nsp13—together with concatenated ICTV exemplar species of each subgenus and other published coronavirus sequences (kindly provided by A. Gorbalenya). These conserved regions are currently used in coronavirus taxonomy for species demarcation with DEmARC software, and for the construction of phylogenetic trees as described in ICTV proposal materials [44]—as described recently for classifying the pandemic SARS-CoV-2 [18]. A maximum likelihood phylogenetic tree was constructed from this alignment (Figure 2). The four genera—i.e., *Alphacoronavirus*, *Betacoronavirus*, *Gammacoronavirus*, and *Deltacoronavirus*—make up the four main clades of the tree. The Danish BtCoV sequences are found within the *Alphacoronavirus* genus, where they form three clusters, consistent with the full-length nucleotide comparison shown in Figure 1. The *M. daubentonii* sequences and the *P. pygmaeus* sequence (from sample 7542-55) form the *M. daubentonii* cluster, which also includes the *Scotophilus* bat coronavirus 512 and porcine epidemic diarrhoea virus—the ICTV exemplar species of the subgenus *Pedacovirus*. In the *P. pygmaeus* sample B40-5 cluster, the exemplar species *Pipistrellus kuhlii* coronavirus 3398 and *Nyctalus velutinus alphacoronavirus* SC-2013 of the subgenus *Nyctacovirus* are present. Finally, the *M. dasycneme* cluster contains the *Myotis ricketti alphacoronavirus* Sax-2011, the single exemplar species of the subgenus *Myotacovirus*.

### 3.2. Bat Astroviruses

Bat astrovirus (BtAstV) reads were identified in the two NGS datasets generated from samples 13585-58 and 21164-6 collected from *M. daubentonii* bats (Table 1 and Table 2). Astroviruses are non-enveloped viruses with a positive-sense single-stranded RNA genome, generally of 6.4–7.9 kb in length [31]. A full-length BtAstV genome for sample 13585-58 of 6557 nucleotides was assembled by mapping to a single de novo generated contig. For sample 21164-6, the SPAdes de novo assembler generated 17 overlapping partial-genome-length astrovirus contigs (Supplementary Table S2). By mapping the reads to these contigs and visually inspecting the assemblies, it was evident that two distinct astroviruses were present in the sample. Through iterative mappings, 2 almost-complete BtAstV genomes of 6619 (A) and 6558 nucleotides (B) with 92.2% identity (corresponding to 516 nucleotide differences between them) were assembled, with different stringency to the de novo contigs. The astrovirus genome is divided into a 5′-untranslated region (UTR), three open reading frames (ORFs)—termed ORF1a, ORF1b, and ORF2—and a 3′-UTR with a poly (A) tail [31]. Although the two BtAstVs from sample 21164-6 were of equal or longer length than the other BtAstV identified, it was not possible to identify the 5′-end of ORF1 or the 3′-end of ORF2 in order to establish the complete coding regions of the genome in sequences A and B. The only full-length reference sequences of the *Mamastrovirus* genus resembling these were the bat astroviruses of 6650 nt (accession number (Acc. No.) MT734809) and 5310 nt (Acc. No. MW249010), and a mouse astrovirus of 6543 nt (Acc. No. JF755422). From alignments with these sequences, it is expected that only a handful of codons are missing from either end of the A sequence. Sequence B is slightly shorter than A and, furthermore, 4 predicted nucleotides at positions 3100–3103 are undefined (marked NNNN) due to low coverage in this region.

BLASTn searches using the 3 assembled genomes (Table 4) showed high similarity (90.2–99.5% identity) to partial ORF1b sequences of 379–381 nt from BtAstVs from *M. daubentonii* bats in Germany [45]. This could also be seen in a phylogenetic tree based on nucleotide sequences of the same partial ORF1b region, as targeted by the most commonly used RT-PCR assay for astrovirus detection [45] (Supplementary Figure S1).

The assembled nucleotide sequences were aligned with the progressive Mauve plugin to search for recombination or other rearrangements between the astrovirus genomes of Danish bats and resembling sequences from BLASTn, as well as ICTV exemplars and additional isolate sequences for each astrovirus of the *Mamastrovirus* genus. The program identified ORF1ab as a common local collinear block (LCB) for the three sequences from Danish bats, although with substantial differences, while ORF2 belonged to different LCBs for the two sequenced samples. No recombination or other rearrangements were detected.

**Figure 3:**
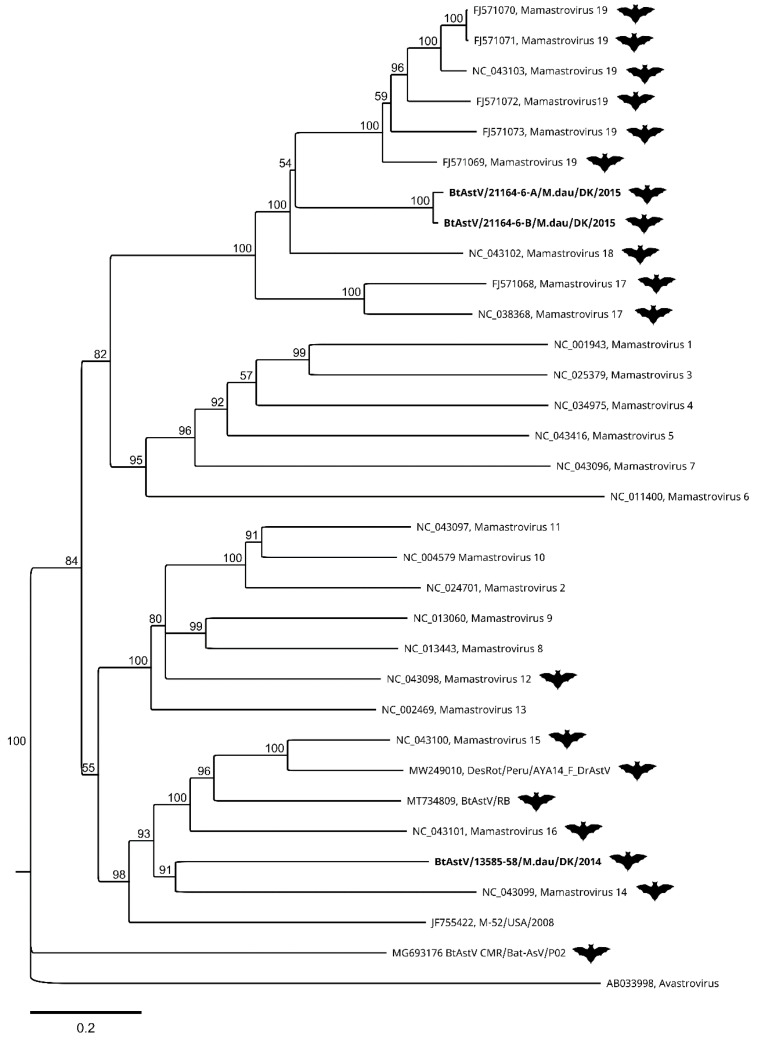
Phylogenetic tree of bat astrovirus sequences from Denmark. The Danish sequences (indicated in bold text) were analysed with the closest matching sequences with full ORF2 coding sequences available in GenBank, and at least one ICTV exemplar isolate of each species of the genus *Mamastrovirus* in the *Astroviridae* family, with an exemplar isolate of the genus *Avastrovirus* chosen as an outgroup. Bat astrovirus sequences are indicated with a bat symbol. The tree was generated using the neighbour-joining method, with 1000 bootstrap iterations on MUSCLE-aligned predicted amino acid sequences from the complete ORF2 gene coding sequence, with a total length of 1144 amino acids. Bootstrap values above 50 are displayed.

The ORF2 coding sequences from the three assembled genomes of BtAstV were translated in silico and aligned with ICTV reference sequences as well as the few, most closely related, astroviruses with full-length ORF2 sequences from GenBank identified by BLAST. A neighbour-joining tree with 1000 bootstrap replicates was constructed with these, in accordance with the proposal from the ICTV [47]. In this tree (Figure 3), the BtAstV from sample 13585-58 forms a cluster with Mamastroviruses 14, 15, and 16, as well as the bat astroviruses and the mouse astrovirus from GenBank. The closest node is shared with Mamastrovirus 14 from *Miniopterus magnater*, but the branch length distance is shorter to Mamastrovirus 15 from *Taphozous melanopogon*. The two BtAstVs from sample 21164-6 are in a different cluster together with Mamastroviruses 17, 18, and 19. The closest node (with a bootstrap value of only 53) is shared with Mamastrovirus 18 from *Miniopterus pusillus*, but again the branch length is shorter to Mamastrovirus 19 from *Taphozous melanopogon*.

### 3.3. Rhopalosiphum Padi Viruses

Many reads (around 780,000) corresponding to sequences from *Rhopalosiphum padi virus* (RhPV) were identified within the dataset from sample OV-157 from *M. daubentonii* (Table 2). RhPV is a member of the family *Dicistroviridae* within the *Picornavirales* order, and has a small monopartite genome of positive-sense single-stranded RNA. The two aphid-infecting dicistroviruses of the genus *Cripavirus*—RhPV, and aphid lethal paralysis virus—both employ plants as passive vectors for their arthropod hosts [48]. A single de novo contig was produced from the RhPV reads in the dataset, but when the reads were mapped to this, it was again evident that two virus variants were present. It was possible to separate the reads of the two RhPVs by strict mapping of all reads to the de novo contig sequence, followed by a less strict mapping of the unused reads (Supplementary Table S2).

As with the other dicistroviruses, the genome of RhPV contains two ORFs preceded by internal ribosomal entry sites (IRESs) directing cap-independent initiation of translation [49]. For the IRES located in the intergenic region (IGR) between ORF1 and ORF2, translation is initiated from a non-AUG codon [50]. ORF1 and ORF2 encode non-structural and structural protein precursors, respectively. The 2 Danish RhPV sequences of 10,009 and 10,010 nucleotides were aligned with a closely related sequence (MF535298) from a bat faecal sample from Hungary identified by BLAST, and with the ICTV exemplar species isolate of RhPV (NC_001874). Nucleotide sequence comparisons were made for each UTR and ORF, as well as for the predicted amino acid (a.a.) sequences encoded by the two ORFs (Table 5). Overall, the similarity of the Danish sequences to the references was high, with 96.5% and 96.4% identity to NC_001874, and 99.5% and 99.2% identity to MF535298, respectively. The greatest difference to the reference sequences was observed in the 5′UTR, where there was 91.9% and 91.7% identity to NC_001874, respectively, but 100% identity to MF535298, although the latter sequence was incomplete.

Interestingly, the 2 Danish nucleotide sequences resemble the Hungarian sequence more than each other (98.9% identity), but both of the Danish sequences have a premature stop codon in the ORF2 due to a frameshift mutation (insertion of U) after nucleotide 9460, which results in the deletion of 30 amino acids (residues 789 to 817). This means that one of the three major structural proteins of RhPV—the 30 kDa capsid protein VP1 [29,50]—will be around 26 kDa instead. A similar premature stop codon in this region has been described in RhPV from wild aphids in Sweden, with no apparent difference in infectivity [52].

### 3.4. Kadipiro Viruses

More than two-thirds (around 2 million) of the trimmed reads in dataset D32-7 from sample 21164-6 from *M. daubentonii* were identified as Kadipiro virus (KDV) reads (Table 2). Kadipiro virus is one of three currently recognized species of virus, along with Banna virus and Liao ning virus, within the genus *Seadornavirus* in the *Reoviridae* family [53]; recently proposed members of this genus are the Balaton and Mangshi viruses [54,55]. Mosquito species are vectors for seadornaviruses, which have been isolated from ticks, encephalitic human patients, pigs, and cattle (Banna virus) [56,57], or are able to infect and kill mice in experimental settings (Liao ning virus) [58]. The genome is double-stranded RNA divided into 12 segments numbered according to size, with a total length of around 21 kbp [53]. The 12 genome segments of KDV were assembled by mapping the reads to the 322 overlapping de novo KDV contigs on a scaffold of the KDV strain JKT-7075 reference sequence from Java, Indonesia, and extracting a consensus sequence. Typically, one long de novo contig covered the majority of the genome segments, while multiple shorter, very varied, de novo contigs mapped to the 5′ and 3′ ends (Supplementary Table S2). For the purposes of this study, it was not necessary to dissect the variants found in the reads and contigs further (Supplementary Material, SPAdes KDV de novo contigs).

The 12 KDV genome segments were subjected to BLASTn searches and compared with the other full-length KDV genome nucleotide sequences available in GenBank (Table 6). The level of identity to the first BLASTn hit varied from 83% (segment 5) to 93% (segment 12, the most conserved segment of KDV), with the closest reference sequence being either strain QTM27331 (marked in red) or strain SDKL1625 (marked in blue) from China.

The VP1 gene, genome segment 1, encodes the RNA-dependent RNA polymerase (RdRp). The predicted amino acid sequence of the RdRp from the assembled KDV was aligned with the amino acid sequences of the closest BLAST matches and ICTV reference sequences, and a maximum likelihood tree with 100 bootstrap replicates was generated (Supplementary Figure S2). Here, it can be seen that the translated Danish KDV shares the closest node with the JKT-7075 reference sequence from Java, but the branch length distances to the Chinese strains are shorter.

### 3.5. Other Viral and Microbial Agents Present in the Data

The online metagenomics analysis tool Kaiju listed many different organisms—including bacteria, viruses, fungi, and parasites—whose nucleic acid sequences available in NCBI databases matched the sequences present in the NGS datasets (Table 2), and some of the virus genomes were fully or partially assembled (Table 3 and Supplementary Table S2).

A full-genome sequence of a polyomavirus from the *M. daubentonii* sample 21164-6 was de novo assembled into a single contig. Polyomaviruses are small, non-enveloped viruses with a circular, double-stranded DNA genome encoding early expressed regulatory genes (encoding large tumour antigen (LTAg) and small tumour antigen (STAg)) and late expressed protein genes [61]. From a phylogenetic analysis of the predicted amino acid sequence of the LTAg (Supplementary Figure S3), the sequence from the Danish bat is most related to two other polyomaviruses identified in bats—Pomona leaf-nosed-bat-associated polyomavirus (Acc. No. NC_033737) [62], and KSA403 polyomavirus isolate from *Eidolon helvum* in Saudi Arabia (Acc. No. NC_040600) [63]—but it is still clearly separate from these, and sequence comparison shows that the Danish sequence only shares 42% and 47% amino acid identity of the LTAg and 57% and 58% full-length genome nucleotide identity with them, respectively. The three bat-derived sequences are situated within a diverse clade of fish polyomaviruses that are unassigned to genera [61,62,63].

Rotavirus H reads were identified in the *M. dasycneme* sample data, and partial genome sequences of 9 out of 11 genome segments were assembled. These resembled sequences from *M. daubentonii* rotavirus H detected in bats in Switzerland in 2019 (e.g., Acc. No. MT815963) [64], with around 93% identity.

The sample from *M. daubentonii*, OV-157, contained calicivirus reads that were de novo assembled into an almost-complete coding sequence, resembling Acc. No. KU712497, with 93% nucleotide identity to a partial-genome bat calicivirus from the same bat species previously described from Hungary [65]; calicivirus reads were also identified in the *M. daubentonii* sample 21164-6, but the three assembled short partial-genome sequences only shared 70–95% identity with the Hungarian reference sequence.

Several different picornaviruses and picorna-like viruses were identified in the samples from *M. daubentonii*, resembling the findings of other metagenomics studies on bats [66]. A picornavirus from sample 13585-58 was partially assembled, and shared 62% nucleotide identity with Washington bat picornavirus from a metagenomics data set (Acc. No. KX580885). This reference sequence is possibly related to a picornavirus identified in a spider [67]. From the *M. daubentonii* sample 21164-6, a full-length genome sequence of the provisionally named Basavirus (bat stool-associated RNA virus) was de novo assembled, with a genome organization resembling the order *Picornavirales*, but not clearly belonging to any of the five established families, therefore inhabiting its own group of Posa and Posa-like viruses (PPLV) [68]. The Danish Basavirus sequenced shared 64% nucleotide identity with the Basavirus 7 isolate 16715_71 from a *Scotophilus kuhlii* bat in Vietnam (Acc. No. KX673238) [68].

The *M. daubentonii* samples also contained many reads from different aphid viruses, e.g., rosy apple aphid virus and *Aphis glycines* virus 2 [69]. A full-length sequence of rosy apple aphid virus was de novo assembled from sample 13585-58, sharing 82% nucleotide identity with the reference sequence (Acc. No. DQ286292) [70]. Furthermore, many reads matched with unclassified RNA viruses described in a large study of newly discovered invertebrate viruses [43].

## 4. Discussion

The faecal samples investigated in this study were initially identified as coronavirus positive by real-time RT-qPCR assays and Sanger sequencing, as used previously by Lazov et al. [27]. The alphacoronaviruses detected in these samples from different bat species were compared using phylogenetic analysis based on short regions (130 or 208 nt) of the ORF1ab coding sequence. Using the nucleic acid samples for NGS sequencing generated 8800–140,000 coronavirus reads for each sample (Table 2). This enabled the generation of 7 full-length BtCoV genome sequences of 27,943–28,192 nucleotides—two of which were from the same sample. The single exception was a sample from *P. pygmaeus* with only 1800 BtCoV reads, which could not be assembled into a full genome, but the partial ORF1ab sequences needed for including it in a standard phylogeny were obtained (Figure 2). Comparison of full-length genome sequences (Figure 1), and the phylogenetic tree of the concatenated conserved predicted amino acid sequences (Figure 2), showed the same species-specific clustering as described in our previous study based on much shorter sequences [27]. The BtCoVs sequenced in this study from *M. daubentonii* are highly similar to one another, and also to a full-length genome sequence from *M. daubentonii* from Finland [28]. The Finnish sample was from Mustasaari on the west coast of Finland, about 1000 km from the sampling site of Mønsted in Denmark. The authors reported that this sequence clustered together with sequences from Central and Southern Europe in a phylogenetic tree of partial ORF1ab nucleotide sequences, which is in line with our previous findings [27]. There is a lack of full-genome coronavirus sequences (or even sequences for the concatenated conserved 1200 amino acid long sequences), from other *M. daubentonii* in GenBank, which prevents more detailed comparisons. The sequences found most closely related to the *M. daubentonii* cluster were from an *M. brandtii* from Finland and the partial-genome sequence from *P. pygmaeus* from 2014, with only around 79.5% nucleotide identity to the first full-length genome sequence (Figure 1). Together, these sequences are part of a larger cluster, which includes the reference sequences for *Scotophilus* bat coronavirus 512 and the porcine epidemic diarrhoea virus (PEDV), suggesting that they all belong to the same subgenus, termed *Pedacovirus*. The *M. dasycneme* BtCoV sequence in this study was assembled from data generated from a sample from Mønsted, as with the *M. daubentonii* samples, but this BtCoV was found to be most closely related—with 79.2% nucleotide identity—to the Chinese reference sequence NC_028811 of the virus species *Myotis ricketti* alphacoronavirus Sax-2011. These sequences also cluster together in the phylogenetic tree (Figure 2), suggesting that the Danish sequence belongs to the subgenus *Myotacovirus*. We saw in our previous study that there were two different types of BtCoV from *P. pygmaeus* in the Danish samples. One of these was the *Pedacovirus*-like partial-genome sequence (as described above). The other type (represented in this study by sample B40-5) is in the same cluster in the phylogeny (Figure 2) as the two species exemplars of the *Nyctacovirus* subgenus: *Nyctalus velutinus* alphacoronavirus SC-2013 and *Pipistrellus kuhlii* coronavirus 3398. Further analysis should establish whether these viruses are prototypes for new coronavirus species, or belong to already established species within the subgenera.

Cell receptor binding is important when it comes to assessing the host species and tissue specificity of potentially zoonotic viruses. The six *Pedacovirus*-like BtCoV sequences described in this study are phylogenetically related to both PEDV and *Scotophilus* bat coronavirus 512. PEDV has been shown to bind to aminopeptidase-N (APN), but not necessarily as the only receptor, while *Scotophilus* bat coronavirus 512 is able to infect a wide range of cultured cell types without the use of APN [71]. To our knowledge, no cell receptors have been described for the *Alphacoronavirus* subgenera *Nyctacovirus* and *Myotacovirus*, but for other subgenera in this genus, APN, angiotensin-converting enzyme 2 (ACE2), N-glycolylneuraminic acid (Neu5Gc), and N-acetylneuraminic acid (Neu5Ac) are known receptors [72]. However, in order to predict the cell receptor specificity of the BtCoVs, sequence similarity studies must be combined with functional studies, as it has previously been shown that only a few amino acid differences in the receptor binding domains can change the receptor specificity completely [73].

This study started out as an attempt to expand the knowledge of coronaviruses in Danish bats. This goal was accomplished and, furthermore, information about various other viruses was obtained as well, and some of their whole-genome sequences could be assembled. Among these were three bat astrovirus genome sequences from two different *M. daubentonii* bats.

Astroviruses of the *Mamastrovirus* genus have been described in many different mammalian species and in many different bats [32]. However, the reference sequences available for the seven species of bat astrovirus currently recognized—namely, *Mamastrovirus 12*, *14*, *15*, *16*, *17*, *18*, and *19*—are only partial genome sequences, mainly covering the ORF1b and ORF2 [39]. In 2020 and 2021, three complete or nearly complete BtAstV genomes (Acc. No. MT734809, MW249010, and MG693176) were released in GenBank, but there is still a lack of full-length sequences from bat astroviruses. The most common approach for sequence comparison and phylogenetic analysis of bat astroviruses is based on the sequence of the PCR product produced by a diagnostic assay [46]. Comparison of the target region of the diagnostic assay within the sequences generated by this study shows that these Danish viruses are most closely related to bat astroviruses detected in bats from Germany (Table 4 and Supplementary Figure S1). The two sequences were found to closely resemble (98.2–99.5% identity) two different sequences of a common cluster of many *M. daubentonii* BtAstVs with different regional origins in Germany [45]. In Figure 3, it can be seen that these two Danish BtAstVs do not clearly belong to either of the species *Mamastrovirus 18* or *19*, but are distantly related to both. The third astrovirus was 93% identical to a German sequence in a distinct cluster with only two sequences [45]. This Danish BtAstV was found to be separate from the rest of the reference sequences (Figure 3), and is distantly related to *Mamastrovirus 14, 15*, and *16*, another bat astrovirus (Acc. No. NC_043101), and mouse astrovirus (Acc. No. JF55422). We therefore propose that this BtAstV is a new, separate species in the *Mamastrovirus* genus. Bat astroviruses are generally regarded as non-pathogenic for bats, but a recent study showed that Jamaican fruit bats, *Artibeus jamaicensis*, experience shifts in their gut microbiome towards increased pathogen-containing microbial genera, and may be more likely to contract co-infections after astrovirus infection [74]. Infections with two or more distinct astroviruses, as seen in one of our bat samples (21164-6), have been documented before—e.g., in a recent Polish study, where 5 out of 12 bats harboured multiple astroviruses, providing an opportunity for the recombination and emergence of new viruses [75]. Cross-species transmission and recombination between astroviruses of different species poses a risk to human and animal health, and is more common than previously thought [76,77].

One of the *M. daubentonii* bats generated reads that could be assembled into a full-length genome of a polyomavirus. More than 100 species of polyomavirus are currently recognized from humans and a wide range of animals, including bats. They are considered to be host-specific, often establishing persistent infections in healthy hosts, and are associated with disease in immunocompromised individuals—e.g., Merkel cell polyomavirus causing Merkel cell carcinoma in humans [61]. However, it is not known whether polyomaviruses can cause disease in bats. Interestingly, the polyomavirus assembled in this study was only distantly related to other previously described viruses, phylogenetically grouping with fish and bat polyomaviruses unassigned to genera (Supplementary Figure S3). The virus was probably present at a high level in the faeces sample, as it was possible to assemble the whole genome in spite of the DNAse I treatment of the purified nucleic acids during the preparation steps for sequencing.

Calicivirus reads were identified from two *M. daubentonii* samples, and one long partial genome and two short sequences were assembled (Table 3). These sequences were identified as belonging to the *Sapovirus* genus of the *Caliciviridae* family, resembling a virus previously described from *M. daubentonii* in Hungary (Acc. No. KU712497) [65]. Sapoviruses cause enteric infections in both humans and animals, and recombination events between sapoviruses of different species have been shown to drive viral evolution [78]. Another important infectious agent is rotavirus, responsible for acute gastroenteritis cases in humans and animals [56]. In this study, we identified reads of rotavirus H in the sample from *M. dasycneme* (Table 2), and assembled partial genome sequences of 9 out of 11 genome segments (Table 3) that most closely resembled rotavirus H sequences from *M. daubentonii* in Switzerland [64].

In addition to the mentioned viruses of vertebrates, we detected numerous reads from viruses specific to invertebrate hosts (Table 2). These viruses included dicistrovirus RhPV, which is capable of infecting aphid species [79]. Other dicistroviruses of the *Cripavirus* genus included Aphid lethal paralysis virus, Cricket paralysis virus, *Drosophila* C virus, and a novel dicistrovirus related to *Drosophila* C virus that has been detected in the blood of fruit bats [80]. The phylogenetic analysis and nucleotide comparison of the two genomes of RhPV from one Danish bat showed that they are very closely related to an RhPV from Hungary described by Zana et al. [51], which was also identified in bat faeces. The genome contains two large ORFs, but is incomplete at the 5′ terminus, where one of the two IRES structures is located. The Danish RhPV sequences supply this missing information, as the 5′ termini of these viruses are not truncated.

Another virus of invertebrates that we detected in the read data in large numbers, presumably following the ingestion of arthropods, was Kadipiro virus. This virus has not been detected in Europe before, but has previously been detected in and isolated from mosquitoes in Asia, and three full-genome sequences are currently available in GenBank from China and Indonesia [30,43,81,82]. Kadipiro virus was detected in a plasma sample from a febrile adult in Kenya, but the results were possibly due to contamination, and could not be verified [59,60]. It is not known whether KDV is able to infect humans or other mammals via mosquito vectors, as seen with Banna virus from the same *Seadornavirus* genus [56]. The 12 segments that make up the viral genome were assembled as consensus sequences, but multiple variants were present in the 2 million reads (Supplementary Material, SPAdes KDV de novo contigs). The assembled genome sequences were compared with the existing reference sequences (Table 6), and the level of nucleotide identity varied from 83 to 93% for segments of the Chinese KDV reference strains QTM27331 and SDKL1625. Phylogenetic comparison of predicted amino acid sequences for the VP1s (RdRPs) showed that the Danish KDV was related to both the Chinese strains and the JKT-7075 isolate from Java, Indonesia, but not clearly to one more than the others (Supplementary Figure S2).

Numerous viruses have been isolated from bat species [9], but some of the most common virus detections in faecal samples from European bats are of astroviruses, coronaviruses, adenoviruses, paramyxoviruses, herpesviruses, and picornaviruses [45,83], with varying prevalence reported—e.g., between 5 and 46% for astroviruses [45,46,84,85], and 2–20% for coronaviruses [25,27,84,85,86]. It is therefore not surprising that individual bats may test positive for several of these viruses, and co-infection of bats with, for example, coronaviruses and astroviruses has previously been reported in 6% of sampled bats in Hong Kong [46] and in one *P. pygmaeus* bat (out of 6 sampled) in a surveillance study in Hungary [85]. Furthermore, out of 17 *Hipposideros cervinus* bats positive for BtCoV, 15 were also found positive for BtAsV in a study in Borneo [84]. Co-infection with different viruses, but especially with positive-sense RNA viruses of the same species, can provide opportunities for recombination [87]. For coronaviruses, recombination has been associated with several documented switches in host and tissue tropism, and is considered to be one of the driving forces of coronavirus evolution [16,24]. In this study, we found multiple examples of virus co-infections, but no evidence of virus recombination in the *Myotis* bats (Table 2 and Table 3). The five bat samples of this genus were included in the study because they scored positive for coronaviruses. Two of the bat samples contained astroviruses as well, and one of the samples—21164-6—actually had two different strains of astrovirus, two different strains of coronavirus, two different strains of calicivirus at low levels, and polyomavirus (Table 1, Table 2 and Table 3). Furthermore, this sample had viral reads from Basavirus and very high levels of KDV reads. However, it is not known whether these viral reads only represented virus ingested with the invertebrate food sources, whether they represented the virome of commensal or parasitic organisms, or whether there was an actual infection in the bat. No other samples—e.g., serum or organs—are available from the bats for further investigations into this potential arbovirus reservoir [88]. Likewise, one of the *M. daubentonii* bat faecal samples contained a high level of RhPV RNA, which may be of little relevance to the bat, but may give insight into the ecological role of bats as disseminators of viral infections of insects [13].

Finding several virus strains in the same sample, some of which were previously uncharacterized—such as the BtAstVs—highlights one of the strengths of the semi-metagenomic sequencing strategy employed in this study, as opposed to amplicon sequencing or other targeted methods. Usually, however, this comes at the cost of lower sequencing coverage of specific targets. Nevertheless, with the exception of one of the *P. pygmaeus* BtCoVs, we have still been able to obtain full-genome sequences of the BtCoV RNA present in six other nucleic acid samples investigated and, thus, the value of this approach is demonstrated.

## Figures and Tables

**Table 1:** Overview of bat faecal sample material used in the study. Previously published data mentioned with GenBank accession numbers consist of partial bat coronavirus ORF1b sequences of 208 or 130 nucleotides in length.

Sample ID	Species	Collection Time	Location	Previous Data	Virus Sequences Assembled in This Study
13585-35	*M. dau*	October 2014	Mønsted	LR025670, LR025709	BtCoV
13585-58	*M. dau*	October 2014	Mønsted	LR025680, LR025712	BtCoV, BtAstV, BtPV, RAAV
21164-6	*M. dau*	November 2015	Mønsted	LR025732	BtCoV, BtAstV, BtCV, BtPyV, KDV, BaV
OV-157	*M. dau*	October 2018	Mønsted	-	BtCoV, BtCV, RhPV
18802-1	*M. das*	November 2016	Mønsted	LR025723	BtCoV, RVH
B40-5	*P. pyg*	August 2013	Sollerup	LR025698, LR025733	BtCoV
7542-55	*P. pyg*	June 2014	Vadum	LR025676, LR025706	BtCoV

Abbreviations of bat species names are: *Myotis daubentonii* (*M. dau*); *Myotis dasycneme* (*M. das*); and *Pipistrellus pygmaeus* (*P. pyg*). Abbreviations of virus names are: bat coronavirus (BtCoV); bat astrovirus (BtAstV); bat calicivirus/sapovirus (BtCV), rotavirus H (RVH); bat polyomavirus (BtPyV); bat picornavirus (BtPV); Basavirus (BaV); Kadipiro virus (KDV); *Rhopalosiphum padi* virus (RhPV); and rosy apple aphid virus (RAAV).

**Table 2:** Overview of the output from Kaiju metagenomics analysis, with a focus on reads assigned to selected viruses. Read numbers highlighted in bold indicate that viral genome sequences were assembled.

NGS Dataset	D28-7	D28-9	D32-7	D32-9	D29-6 + D32-10	D32-6	D32-4
Bat species	*M. dau*	*M. dau*	*M. dau*	*M. dau*	*M. das*	*P. pyg*	*P. pyg*
Sample no.	**13585-35**	**13585-58**	**21164-6**	**OV-157**	**18802-1**	**B40-5**	**7542-55**
Biosample ac. no.	SAMN 18653548	SAMN 18653642	SAMN 18661215	SAMN 18680226	SAMN 18665528	SAMN 18672062	SAMN 18672089
Assigned reads (% of raw data)	296,356 (23%)	1,469,272 (42%)	2,234,894 (63%)	2,155,628 (62%)	1,495,375 (49%)	55,361 (19%)	182,572 (50%)
*Archaea*	2221	503	132	352	2078	207	360
*Bacteria*	189,602	1,121,102	28,900	164,552	1,447,060	39,417	148,103
*Eukaryota*	22,941	144,898	8868	18,813	18,251	2438	9956
**Viruses:**	72,440	189,014	2,195,308	1,961,910	8998	10,187	2871
Unclassified vir.	13,181 ^1^	7865 ^1^	**3742** ^2^	497,671 ^1^	6	8	3
Unclas. RNA vir. [43]	44,631 ^3^	40,180 ^3^	6584 ^4^	654,384 ^3^	264	401	5
Unclas. bacterial vir.	26	2997	-	13	22	8	20
Retro-transcribing vir.	386	44	31	66	276	113	115
dsDNA viruses	1012	208	**233** ^5^	97	1565	84	188
ssDNA viruses	-	23	-	-	16	7	-
dsRNA viruses	458 ^6^	29	**2,113,874** ^7^	770 ^8^	**90** ^9^	-	-
**ssRNA viruses:**	9980	136,492	70,512	734,899	6708	9501	2517
Unclassified vir.	22	**7242** ^10^	-	306	-	-	-
*Coronavirinae*	**6381**	**117,972**	**68,838**	**52,185**	**5934**	**7273**	**1173**
*Astroviridae*	8	**5754**	**744**	2	-	-	-
*Tymovirales*	87	2811	54	15	7	191	-
*Nodaviridae*	8	-	357	-	6	-	-
***Picornavirales:***	3166	1094	435 ^13^	681,091	41	1825	14
*Picornaviridae*	-	**902** ^12^	-	42	-	2	-
*Iflaviridae*	443	137	-	5063 ^15^	12	1795 ^14^	-
*Dicistroviridae*	2679 ^11^	17	48	**675,984** ^11^	26	19	10
*Caliciviridae*	-	-	**28**	**276**	-	-	-

^1^ Predominant species: *Aphis glycines* virus 2; ^2^ predominant species: Basavirus sp.; ^3^ predominant species: Hubei permutotetra-like virus 4; ^4^ predominant species: Hubei partiti-like virus 22; ^5^ predominant species: Pomona leaf-nosed-bat-associated polyomavirus; ^6^ predominant species: unclassified Birnaviridae; ^7^ predominant species: Kadipiro virus; ^8^ predominant species: Totiviridae; ^9^ predominant species: rotavirus; ^10^ predominant species: rosy apple aphid virus; ^11^ predominant species: *Rhopalosiphum padi* virus; ^12^ predominant species: Washington bat picornavirus; ^13^ predominant species: Pow Burn virus; ^14^ predominant species: iflavirus; ^15^ predominant species: *Brevicoryne brassicae* picorna-like virus. Abbreviations used: viruses (vir.); unclassified (unclas.); double-stranded (ds); single-stranded (ss).

**Table 3:** List of all full-length (highlighted in bold) and partial genome sequences assembled in this study and deposited in GenBank.

Virus Description	Virus Strain Name	Mapped Reads	Average Coverage	Length nt.	Accession Number
Alphacoronavirus	BtCoV/13585-35/M.dau/DK/2014	8800	50	**28,096**	MN535731
	BtCoV/13585-58/M.dau/DK/2014	140,000	1000	**28,140**	MN535732
	BtCoV/21164-6/M.dau/DK/2015	70,000	370	**28,117**	MN482243
	BtCoV/21164-6-alt/M.dau/DK/2015	20,000	120	**28,092**	MZ218052
	BtCoV/OV-157/M.dau/DK/2018	13,000	102	**28,192**	MN535733
	BtCoV/18802-1/M.das/DK/2016	9800	42	**28,013**	MN535734
	BtCoV/B40-5/P.pyg/DK/2013	9200	59	**27,943**	MN482242
	BtCoV/7542-55/P.pyg/DK/2014	1800	8	27,786 ^1^	MZ218060
Mamastrovirus	BtAstV/13585-58/M.dau/DK/2014	17,000	465	**6557**	MN832787
	BtAstV/21164-6-A/M.dau/DK/2015	630	15	6619	MZ218053
	BtAstV/21164-6-B/M.dau/DK/2015	540	13	6558 ^2^	MZ218054
Calicivirus	BtCV/OV-157/M.dau/DK/2018	352	8	7486 ^3^	MZ218056
	BtCV/21164-6-A/M.dau/DK/2015	2	1.5	396	MZ218057
	BtCV/21164-6-B/M.dau/DK/2015	6	1.4	706	MZ218058
Rotavirus H	RVH/18802-1/M.das/DK/2016	98	2.2	58–2169 ^4,5^	MZ218062-
					MZ218070
Polyomavirus	BtPyV/21164-6/M.dau/DK/2015	710	17	**4758**	MZ218055
Picornavirus	BtPV/13585-58/M.dau/DK/2014	870	16	9300	MZ218061
Basavirus	BaV/21164-6/M.dau/DK/2015	8560	150	**9155**	MW929926
Kadipiro virus	KDV/21164-6/M.dau/DK/2015	1,960,000	3700–16,500	**754–3750** ^6^	MN543741,
					MN543742,
					MN543744-
					MN543753
*Rhopalosiphum padi* virus	RhPV/OV-157/M.dau/DK/2018	440,000	6490	**10,009**	MN535735
	RhPV/OV-157-alt/M.dau/DK/2018	340,000	4600	**10,010**	MZ218059
Rosy apple aphid virus	RAAV/13585-58/M.dau/DK/2014	5860	102	**9742**	MW929927

Some sequences contain NNNs to indicate unknown nucleotides. Numbers of NNNs are: ^1^ 2037; ^2^ 4; ^3^ 70; and ^4^ 2558. ^5^ A total of 9 out of 11 partial genome segments, with a total length of 5542 nt (excluding NNNs), were assembled into gapped sequences for rotavirus H using both reference assemblies and de novo assembly. ^6^ All 12 genome segments of Kadipiro virus were assembled with a total length of 20,781 nt.

**Table 4:** The closest matching sequences to the bat astroviruses found in this study are not complete genomes, but partial ORF1ab gene sequences generated by sequencing of RT-PCR products (around 381 nucleotides in length without primers) from the most commonly used assay for detection of astroviruses in bats [46]. This table summarizes the top three identity BLASTn search hits of these short sequences as accession numbers, with % identity/length in parentheses. The 1st BLASTn hits were described previously [45]. Abbreviations used for German regions: North Rhine-Westphalia (NRW); and Mecklenburg-Western Pomerania (MV).

BtAstV Genomes	1st BLASTn Hit	1st BLASTn Hit Source	2nd BLASTn Hit	3rd BLASTn Hit
BtAstV/13585-58/M.dau/DK/2014	KT894882 (93.7/379)	*M. daubentonii*, D15, September 2014, NRW	EU847161 (77.5/240)	EU847160 (77.5/240)
BtAstV/21164-6-A/M.dau/DK/2015	KT894889 (98.2/381)	*M. daubentonii*, D13, September 2014, NRW	KT894883 (91.9/381)	KT894893 (92.0/375)
BtAstV/21164-6-B/M.dau/DK/2015	KT894894 (99.5/381)	*M. daubentonii*, D10, July 2013, MV	KT894896 (99.2/381)	KT894895 (99.2/381)

**Table 5:** *Rhopalosiphum padi* virus comparisons to one another, to the reference RhPV sequence NC_001874, and to the closely related sequence MF535298 from Hungary [51].

	RhPV/OV-157/M.dau/DK/2018		RhPV/OV-157-alt/M.dau/DK/2018	
Nucleotide Comparison	(10,009 nt)		(10,010 nt)	
	Nt. Differences	Percent Identity	Nt. Differences	Percent Identity
**NC_001874** (10,011 nt)	351	96.5	357	96.4
- 5′-end (579 nt)	47	91.9	48	91.7
- ORF1 (5997 nt)	230	96.2	231	96.2
- IGR (533 nt)	8	98.5	11	97.9
- ORF2 (2451 nt)	59	97.6	59	97.6
- 3′-end (451 nt)	7	98.4	8	98.2
**MF535298** (9966 nt)	50	99.5	76	99.2
- 5′-end (219 nt)	0	100	0	100
- ORF1 (5997 nt)	19	99.7	48	99.2
- IGR (533 nt)	2	99.6	3	99.4
- ORF2 (2451 nt)	23	99.1	21	99.1
- 3′-end (451 nt)	6	98.7	4	99.1
**OV-157-alt** (10,010 nt)	106	98.9		
- 5′-end (579 and 580 nt)	2	99.7		
- ORF1 (5997 nt)	59	99.0		
- IGR (533 nt)	5	99.1		
- ORF2 (2361 nt)	31	98.7		
- 3′-end (539 nt)	9	98.3		
Amino acid comparison: ORF1	(1988 a.a.)		(1988 a.a.)	
	A.a. differences	Percent identity	A.a. differences	Percent identity
**NC_001874** (1988 a.a.)	23	98.9	28	98.6
**MF535298** (1988 a.a.)	3	99.9	4	99.8
**OV-157-alt** (1988 a.a.)	7	99.7		
Amino acid comparison: ORF2	(788 a.a.)		(788 a.a.)	
	A.a. differences	Percent identity	A.a. differences	Percent identity
**NC_001874** (818 a.a.)	11	98.6	13	98.4
**MF535298** (818 a.a.)	6	99.2	6	99.2
**OV-157-alt** (788 a.a.)	4	99.5		

**Table 6:** Assembled Kadipiro virus genome segments listed with the first three BLASTn search hits as accession numbers, with percent identities in parentheses. KX884650–KX884661: Kadipiro virus strain QTM27331 from China 2013; MG590140–MG590148, MG590150–MG590151: Kadipiro virus strain SDKL1625 from China 2016; AF133429, AF133509–AF133513, AF052019–AF052023: Kadipiro virus strain JKT-7075, an isolate originally from Java, Indonesia [30]; KX247778: Kadipiro virus isolate Kenya2 [59,60]; FJ159105: Kadipiro virus strain YN0557 from China 2005.

Genome Segment	Segment Length	Accession Number	1st BLASTn Hit	2nd BLASTn Hit	3rd BLASTn Hit
Segment 1	3799	MN543741	KX884650 (86)	MG590148 (86)	AF133429 (84)
Segment 2	3016	MN543742	MG590140 (85)	KX884651 (84)	AF134509 (82)
Segment 3	2353	MN543744	MG590141 (84)	KX884652 (84)	AF134510 (81)
Segment 4	2121	MN543745	KX884653 (86)	AF134511 (84)	KX247778 (74)
Segment 5	1895	MN543746	MG590142 (83)	KX884654 (83)	AF134512 (81)
Segment 6	1659	MN543747	KX884655 (87)	AF134513 (86)	MG590143 (86)
Segment 7	1232	MN543748	MG590144 (92)	KX884656 (92)	AF052023 (90)
Segment 8	1083	MN543749	KX884657 (88)	AF052022 (85)	MG590145 (86)
Segment 9	1041	MN543750	KX884658 (91)	MG590150 (90)	AF052021 (88)
Segment 10	914	MN543751	KX884659 (87)	MG590151 (86)	AF052020 (84)
Segment 11	875	MN543752	KX884660 (85)	AF052019 (83)	MG590146 (83)
Segment 12	754	MN543753	MG590147 (93)	FJ159105 (90)	KX884661 (89)

## Data Availability

The data presented in this study are openly available in the NCBI GenBank at https://www.ncbi.nlm.nih.gov/, Bioproject reference number PRJNA720531.

## Supplementary Materials

The following are available online at https://www.mdpi.com/article/10.3390/v13061073/s1: Figure S1: Phylogenetic tree of the partial ORF1b of the bat astroviruses from Denmark; Figure S2: Phylogenetic tree of the Kadipiro virus sequence from Denmark; Figure S3: Phylogenetic tree of the polyomavirus sequence from Denmark; Table S1: Quality control of libraries and reads; Table S2: Additional assembly data; File: SPAdes KDV de novo contigs.
