# Correction: Lazov et al. Full-Genome Sequences of Alphacoronaviruses and Astroviruses from Myotis and Pipistrelle Bats in Denmark. *Viruses* 2021, *13*, 1073

**DOI:** 10.3390/v17101328

**Published:** 2025-09-30

**Authors:** Christina M. Lazov, Graham J. Belsham, Anette Bøtner, Thomas Bruun Rasmussen

**Affiliations:** 1 Department of Biotechnology and Biomedicine, Technical University of Denmark, 2800 Kongens Lyngby, Denmark; 2 Department of Veterinary and Animal Sciences, University of Copenhagen, 1870 Frederiksberg, Denmark; grbe@sund.ku.dk (G.J.B.);; 3 Department of Virus and Microbiological Special Diagnostics, Statens Serum Institut, 2300 Copenhagen, Denmark

**Error in Table**

In the original publication [1], there was a mistake in Table 3 as published. In the table, the list of published genomes of bat coronavirus (BtCoV) stated that the GenBank accession number of strain BtCoV/21164-6/M.dau/DK/2015 was MN543743, but the correct number is MN482243. The corrected version of Table 3 appears below.

**Table 3:** List of all full-length (highlighted in bold) and partial genome sequences assembled in this study and deposited in GenBank.

Virus Description	Virus Strain Name	Mapped Reads	Average Coverage	Length nt.	Accession Number
Alphacoronavirus	BtCoV/13585-35/M.dau/DK/2014	8800	50	**28,096**	MN535731
	BtCoV/13585-58/M.dau/DK/2014	140,000	1000	**28,140**	MN535732
	BtCoV/21164-6/M.dau/DK/2015	70,000	370	**28,117**	MN482243
	BtCoV/21164-6-alt/M.dau/DK/2015	20,000	120	**28,092**	MZ218052
	BtCoV/OV-157/M.dau/DK/2018	13,000	102	**28,192**	MN535733
	BtCoV/18802-1/M.das/DK/2016	9800	42	**28,013**	MN535734
	BtCoV/B40-5/P.pyg/DK/2013	9200	59	**27,943**	MN482242
	BtCoV/7542-55/P.pyg/DK/2014	1800	8	27,786 ^1^	MZ218060
Mamastrovirus	BtAstV/13585-58/M.dau/DK/2014	17,000	465	**6557**	MN832787
	BtAstV/21164-6-A/M.dau/DK/2015	630	15	6619	MZ218053
	BtAstV/21164-6-B/M.dau/DK/2015	540	13	6558 ^2^	MZ218054
Calicivirus	BtCV/OV-157/M.dau/DK/2018	352	8	7486 ^3^	MZ218056
	BtCV/21164-6-A/M.dau/DK/2015	2	1.5	396	MZ218057
	BtCV/21164-6-B/M.dau/DK/2015	6	1.4	706	MZ218058
Rotavirus H	RVH/18802-1/M.das/DK/2016	98	2.2	58–2169 ^4,5^	MZ218062-
					MZ218070
Polyomavirus	BtPyV/21164-6/M.dau/DK/2015	710	17	**4758**	MZ218055
Picornavirus	BtPV/13585-58/M.dau/DK/2014	870	16	9300	MZ218061
Basavirus	BaV/21164-6/M.dau/DK/2015	8560	150	**9155**	MW929926
Kadipiro virus	KDV/21164-6/M.dau/DK/2015	1,960,000	3700–16,500	**754–3750** ^6^	MN543741,
					MN543742,
					MN543744-
					MN543753
*Rhopalosiphum padi* virus	RhPV/OV-157/M.dau/DK/2018	440,000	6490	**10,009**	MN535735
	RhPV/OV-157-alt/M.dau/DK/2018	340,000	4600	**10,010**	MZ218059
Rosy apple aphid virus	RAAV/13585-58/M.dau/DK/2014	5860	102	**9742**	MW929927

Some sequences contain NNNs to indicate unknown nucleotides. Numbers of NNNs are: ^1^ 2037; ^2^ 4; ^3^ 70; and ^4^ 2558. ^5^ A total of 9 out of 11 partial genome segments, with a total length of 5542 nt (excluding NNNs), were assembled into gapped sequences for rotavirus H using both reference assemblies and de novo assembly. ^6^ All 12 genome segments of Kadipiro virus were assembled with a total length of 20,781 nt.

The authors state that the scientific conclusions are unaffected. This correction was approved by the Academic Editor. The original publication has also been updated.
