# Supplementary material for: Full-Genome Sequences of Alphacoronaviruses and Astroviruses from Myotis and Pipistrelle Bats in Denmark
Source: Viruses. 2021 Jun 4;13(6):1073. doi: 10.3390/v13061073 (PMC8229204; doi:10.3390/v13061073)
Supplement: Supplementary file 1 [file viruses-13-01073-s001.zip › viruses-1219303- supplementary material.pdf]

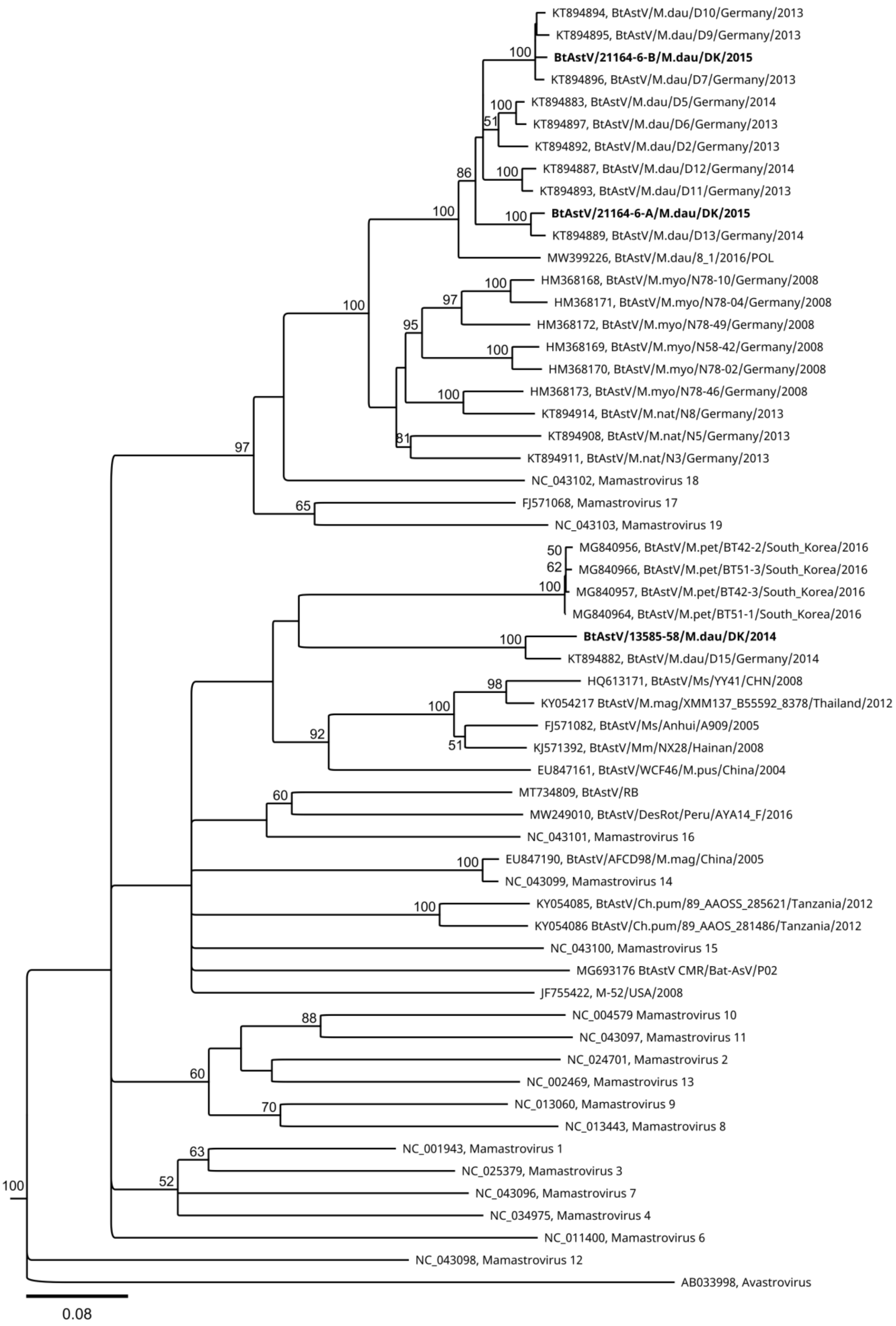

Figure S1. Phylogenetic tree including the three bat astrovirus sequences from Denmark. From these sequences, the target region in ORF1b for the most commonly used astrovirus screening assay [46] was aligned with the closest related nucleotide sequences of minimum length 379 nt identified by BLASTn as well as the same reference sequences as used in Figure 3. The tree was generated using the neighbor-joining method with the Jukes-Cantor genetic distance model and 1000 bootstrap replicates. Nodes with bootstrap support above 30 are shown and nodes with above 50 support are indicated with numbers.

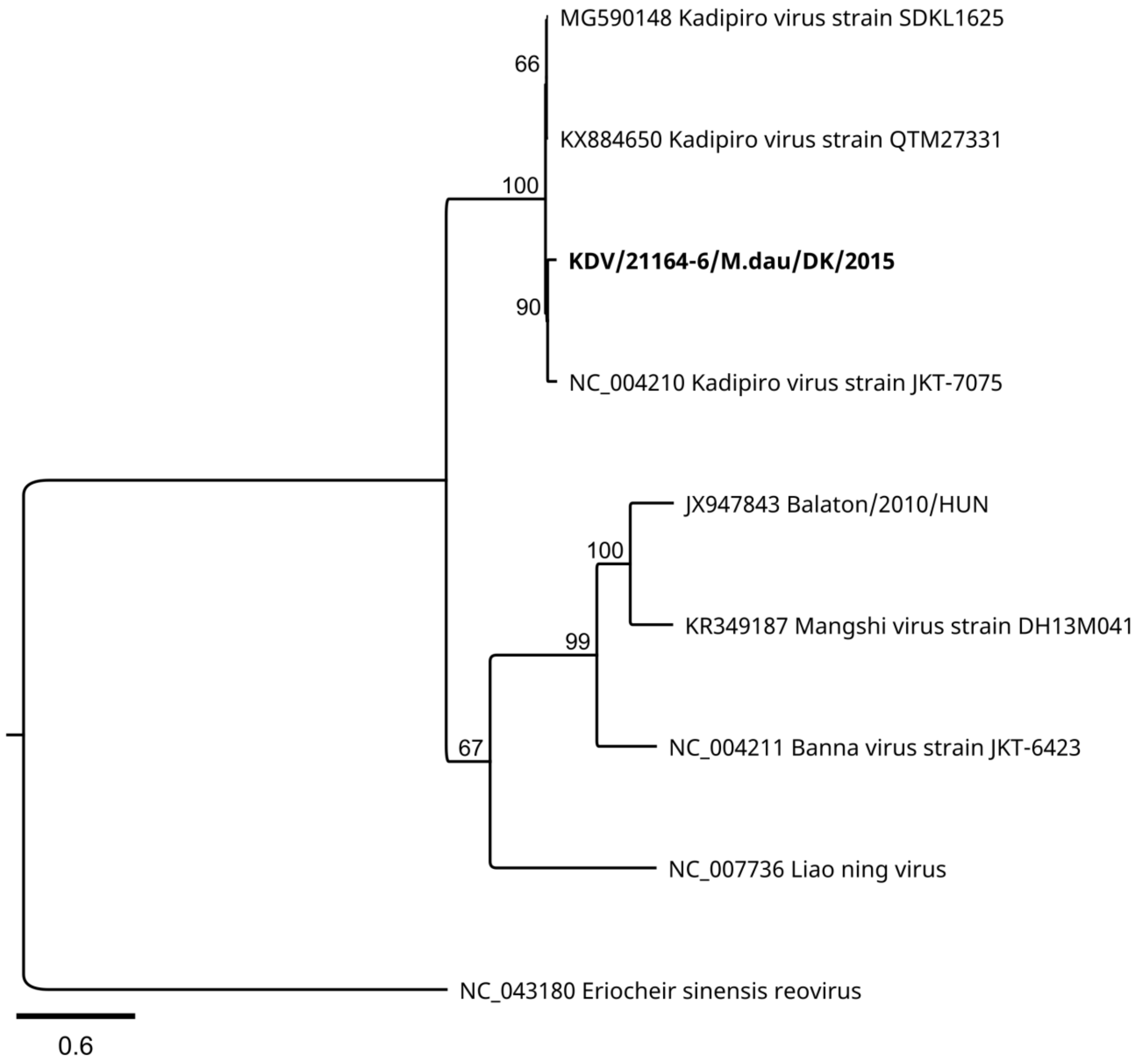

Figure S2. Phylogenetic tree including the Kadipiro virus sequence from Denmark. These sequences were analysed with the closest matching sequences from GenBank and one ICTV exemplar isolate of each species of the *Seadornavirus* genus, subfamily *Sedoreovirinae* with an exemplar isolate of the *Cardoreovirus* genus, *Eriocheir sinensis reovirus,* chosen as an outgroup. Other proposed species of the *Seadornavirus* genus were also included in the tree. These are Balaton virus, sequenced from the intestinal contents of freshwater carp in Hungary [54] and Mangshi virus isolated from mosquitos in China [55]. The tree was generated using the maximum likelihood method, model LG+G with 100 bootstrap iterations on MUSCLE aligned predicted amino acid sequences for VP1 derived from the Kadipiro virus genome segment 1 and corresponding segments of the reference sequences with a total average length of 1,200 amino acids.

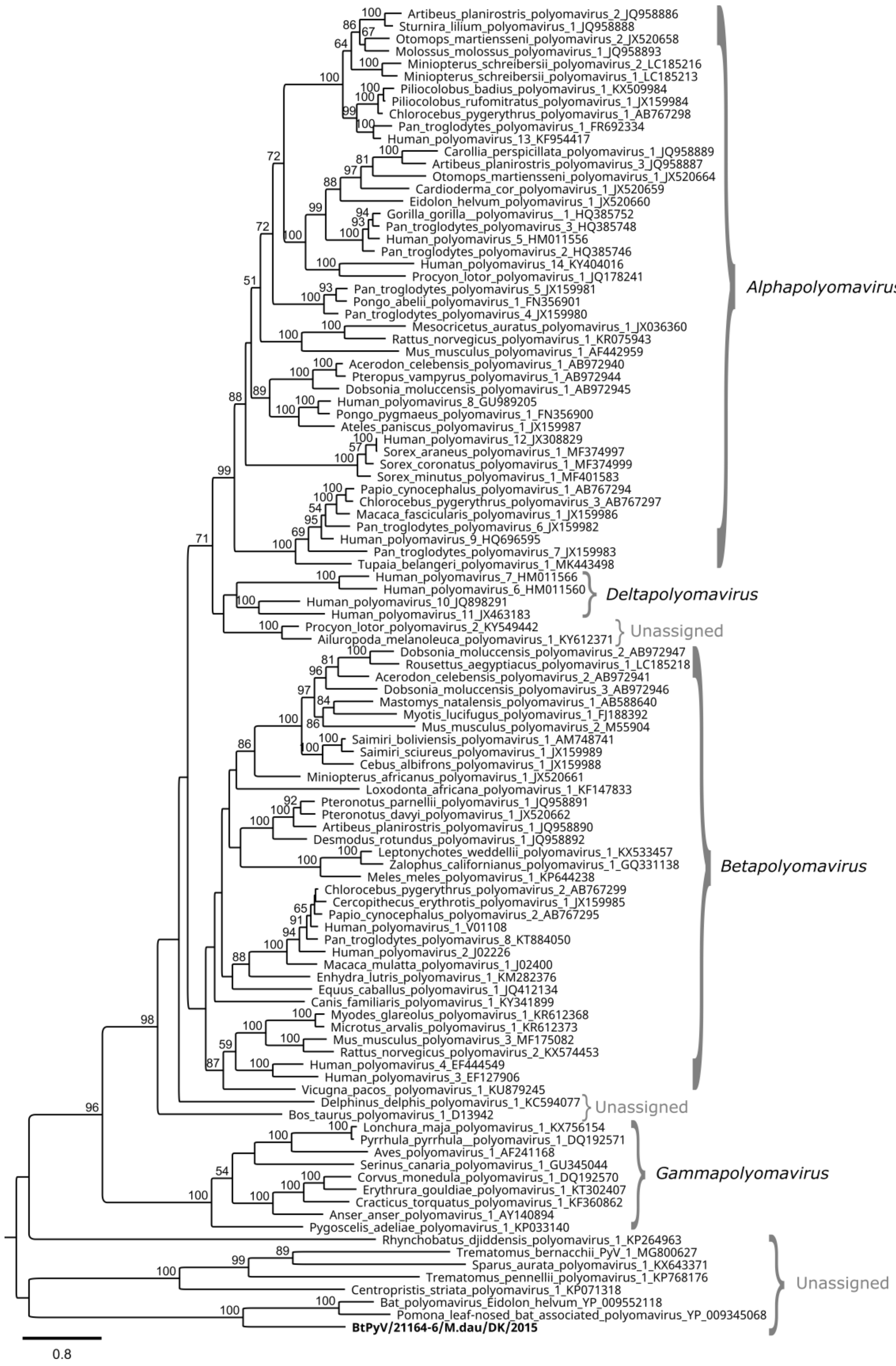

Figure S3. Phylogenetic tree including the polyomavirus sequence from Denmark (marked in bold in the lower end of the tree). The sequence was analysed with the two closest resembling sequences from GenBank using BLAST and ICTV exemplar isolate sequences of each species in the *Polyomaviridae* family obtained from the resources pages of the *Polyomaviridae* figure 3 by ICTV [56]. The currently recognized genera of *Alphapolyomavirus*, *Betapolyomavirus*, *Gammapolyomavirus* and *Deltapolyomavirus* are indicated on the figure. The tree was generated using the maximum likelihood method with 100 bootstrap iterations on MUSCLE aligned predicted amino acid sequences of the LTAg with an average length of 772 amino acids. The evolutionary model for the tree was LG + G. Bootstrap support values above 50 are displayed on the figure.

Table S1. Summary of quality control (QC) parameters for the DNA libraries and NGS datasets, as given by Bioanalyzer 2100 (Agilent Technologies, Glostrup, Denmark) output and the FastQC version 0.11.9 program [34], respectively. Two libraries were generated and sequenced from the sample 18802-1 from *M. dasycneme*. FastQC gives visual information on the parameters mentioned in the table and rates them with the symbols √ (ok), ! (warning) and X (failure). After trimming, some parameters were still rated as failure. These were "Per tile sequence quality" meaning that some areas of the flowcell have worse quality than the average, "Per base sequence content" which is a typical failure reason for RNA-sequencing libraries, "Per sequence GC content", where failure may arise due to contaminants in the library (adapter dimers or different species) and "Sequence duplication levels" with high levels indicating enrichment bias [34].

**NGS library**	**D28-7**	**D28-9**	**D32-7**	**D32-9**	**D29-6 / D32-10**	**D32-6**	**D32-4**
Bat species	*M. dau*	*M. dau*	*M. dau*	*M. dau*	*M. das*	*P. pyg*	*P. pyg*
Sample no.	**13585-35**	**13585-58**	**21164-6**	**OV-157**	**18802-1**	**B40-5**	**7542-55**
Bioanalyzer electropherogram region 200-1000 bp	Low peak 200-300*	No peak*	High, sloping peak 200	High, wide peak 600	Peak 190 / low peak 200	No peak	Low, wide peak 250
Average size (bp)	-	-	338	474	280 / 321	558	331
Concentration (pg/µl)	-	-	1488	1648	248 / 132	20	76
Molarity (pmol/l)	-	-	7645	6541	1425 / 701	71	406
Short read archive acc. no.	SRR 14195149	SRR 14195380	SRR 14195385	SRR 14208005	SRR14195384 / 14195383	SRR 14195382	SRR 14195381
FastQC (raw reads)	1,265,226	3,507,280	3,448,636	3,468,262	2,740,900 / 318,496	295,984	361,798
Average length (range)	130 (35-301)	161 (35-301)	116 (35-301)	176 (35-301)	99 / 151 (35-301)	171 (35-301)	120 (35-301)
%GC	45	43	38	47	57 / 39	43	49
Per base sequence quality	!	X	X	X	X	X	X
Per tile sequence quality	√	!	√	√	! / √	√	√
Per sequence quality scores	√	√	√	√	√	√	√
Per base sequence content	X	X	X	X	X	!	X
Per sequence GC content	√	X	X	!	X / !	X	!
Per base N content	√	√	√	√	√	√	√
Sequence length distrib.	!	!	!	!	!	!	!
Sequence duplication levels	√	X	X	X	√	!	√
Overrepresented sequences	X	!	X	!	X	X	X
Adapter content	√	√	√	√	√	√	√
FastQC (trimmed reads)	935,570	3,186,628	2,861,952	3,149,088	2,000,524 / 207,074	251,950	334,012
Average length (range)	130 (50-296)	157 (50-301)	120 (50-301)	157 (50-301)	111 / 120 (50-301)	153 (50-301)	107 (35-301)
%GC	45	43	38	47	58 / 45	45	50
Per base sequence quality	√	√	√	√	√	√	√
Per tile sequence quality	X	!	X	X	X	X	X
Per sequence quality scores	√	√	√	√	√	√	√
Per base sequence content	!	X	X	X	! / X	X	X
Per sequence GC content	√	X	√	!	X	!	√
Per base N content	√	√	√	√	√	√	√
Sequence length distrib.	!	!	!	!	!	!	!
Sequence duplication levels	√	X	X	X	√	!	√
Overrepresented sequences	√	!	!	√	√	!	!
Adapter content	√	√	√	√	√	√	√

* For the Bioanalyzer run that included testing of the NGS libraries of these two samples, no concentrations were calculated due to a faulty calibration.

Table S2. Additional information about viral assemblies expanding Table 3. Often a combination of both *de novo* assemblies and reference assemblies were used in order to have a scaffold for the *de novo* generated contigs and to map additional viral reads.

Virus strain name	*De novo* contigs	Average *de novo* contig length (range)	Assemblies	Average read length (range)	GC content
BtCoV/13585-35/M.dau/DK/2014	1	28,120	*De novo*	152 (50-296)	41.6 %
BtCoV/13585-58/M.dau/DK/2014	1	28,175	*De novo*	182 (50-301)	41.7 %
BtCoV/21164-6/M.dau/DK/2015	2	14,086 (7762-20,410)	*De novo* + Reference	157 (50-301)	41.4 %
BtCoV/21164-6-alt/M.dau/DK/2015	3	10,727 (4010-20,410)	*De novo* + Reference	152 (50-301)	44.2 %
BtCoV/OV-157/M.dau/DK/2018	1	28,177	*De novo*	173 (50-301)	41.5 %
BtCoV/18802-1/M.das/DK/2016	1	27,852	*De novo*	125 (50-301)	41.2 %
BtCoV/B40-5/P.pyg/DK/2013	1	27,949	*De novo*	171 (50-301)	44.7 %
BtCoV/7542-55/P.pyg/DK/2014	20	1353 (393-4849)	*De novo* + Reference	126 (35-295)	41.9 %
BtAstV/13585-58/M.dau/DK/2014	1	6575	*De novo* + Reference	179 (50-301)	47.0 %
BtAstV/21164-6-A/M.dau/DK/2015	11	589 (196-1320)	*De novo* + Reference	154 (51-296)	47.8 %
BtAstV/21164-6-B/M.dau/DK/2015	8	768 (271-2478)	*De novo* + Reference	154 (50-284)	48.3 %
BtCV/OV-157/M.dau/DK/2018	3	2456 (1276-3888)	*De novo* + Reference	166 (50-298)	51.0 %
BtCV/21164-6-A/M.dau/DK/2015	1	396	*De novo*	300 (300-301)	50.0 %
BtCV/21164-6-B/M.dau/DK/2015	0	-	Reference	163 (52-301)	48.9 %
RVH/18802-1/M.das/DK/2016	1	406	*De novo* + Reference	122 (53-293)	34.9 %
BtPyV/21164-6/M.dau/DK/2015	1	4813	*De novo*	117 (50-269)	49.1 %
BtPV/13585-58/M.dau/DK/2014	2	4690 (2384-6996)	*De novo* + Reference	174 (50-301)	44.2 %
BaV/21164-6/M.dau/DK/2015	1	9155	*De novo*	160 (50-301)	40.3 %
KDV/21164-6/M.dau/DK/2015	322	148 (56-2792)	*De novo* + Reference	105 (50-301)	36.5 %
RhPV/OV-157/M.dau/DK/2018	1	9999	*De novo*	150 (50-301)	38.4 %
RhPV/OV-157-alt/M.dau/DK/2018			*De novo*	144 (50-301)	38.0 %
RAAV/13585-58/M.dau/DK/2014	1	9709	*De novo*	169 (50-301)	38.0 %
